# The Metagenomic Composition and Effects of Fecal-Microbe-Derived Extracellular Vesicles on Intestinal Permeability Depend on the Patient’s Disease

**DOI:** 10.3390/ijms24054971

**Published:** 2023-03-04

**Authors:** Cristina Rodríguez-Díaz, Flores Martín-Reyes, Bernard Taminiau, Ailec Ho-Plágaro, Raquel Camargo, Felix Fernandez-Garcia, José Pinazo-Bandera, Juan Pedro Toro-Ortiz, Montserrat Gonzalo, Carlos López-Gómez, Francisca Rodríguez-Pacheco, Dámaris Rodríguez de los Ríos, Georges Daube, Guillermo Alcain-Martinez, Eduardo García-Fuentes

**Affiliations:** 1 Instituto de Investigación Biomédica de Málaga y Plataforma en Nanomedicina—IBIMA Plataforma BIONAND, 29590 Malaga, Spain; 2 UGC de Aparato Digestivo, Hospital Universitario Virgen de la Victoria, 29010 Malaga, Spain; 3 Facultad de Medicina, Universidad de Málaga, 29010 Malaga, Spain; 4 Fundamental and Applied Research for Animals & Health (FARAH), Department of Food Microbiology, Faculty of Veterinary Medicine, University of Liège, 4000 Liège, Belgium; 5 UCG de Endocrinología y Nutrición, Hospital Regional Universitario, 29009 Malaga, Spain; 6 Centro de Investigación Biomédica en Red de Enfermedades Hepáticas y Digestivas (CIBERehd), 29010 Malaga, Spain

**Keywords:** microbiome, metagenome, fecal-microbe-derived extracellular vesicles, intestinal permeability, diarrhea, morbid obesity, inflammatory bowel disease

## Abstract

The composition and impact of fecal-microbe-derived extracellular vesicles (EVs) present in different diseases has not been analyzed. We determined the metagenomic profiling of feces and fecal-microbe-derived EVs from healthy subjects and patients with different diseases (diarrhea, morbid obesity and Crohn’s disease (CD)) and the effect of these fecal EVs on the cellular permeability of Caco-2 cells. The control group presented higher proportions of *Pseudomonas* and *Rikenellaceae_RC9_gut_group* and lower proportions of *Phascolarctobacterium*, *Veillonella* and *Veillonellaceae_ge* in EVs when compared with the feces from which these EVs were isolated. In contrast, there were significant differences in 20 genera between the feces and EV compositions in the disease groups. *Bacteroidales* and *Pseudomonas* were increased, and *Faecalibacterium*, *Ruminococcus*, *Clostridium* and *Subdoligranum* were decreased in EVs from control patients compared with the other three groups of patients. *Tyzzerella*, *Verrucomicrobiaceae*, *Candidatus_Paracaedibacter* and *Akkermansia* were increased in EVs from the CD group compared with the morbid obesity and diarrhea groups. Fecal EVs from the morbid obesity, CD and, mainly, diarrhea induced a significant increase in the permeability of Caco-2 cells. In conclusion, the metagenomic composition of fecal-microbe-derived EVs changes depending on the disease of the patients. The modification of the permeability of Caco-2 cells produced by fecal EVs depends on the disease of the patients.

## 1. Introduction

Gram-positive and Gram-negative bacteria release membrane vesicles with sizes ranging from 20 to 400 nm in different abundances, structures, and molecular cargo [1,2]. These microbial extracellular vesicles (EVs) represent a secretion and transport mechanism for carbohydrates, lipids and several cell wall components as well as proteins, DNA, RNA and signaling molecules, among others [3]. Therefore, EVs have been related to cell-to-cell communication, virulence, horizontal gene transfer or phage infection [4,5]. Although the outer membrane vesicles (OMVs) of Gram-negative bacteria were the first found and described, recent work has demonstrated the production of other types of EVs by both Gram-positive and Gram-negative bacteria and even mycobacteria and fungi [4]. The types and origins of these EVs were summarized in a previous review, including OMVs, outer-inner membrane vesicles (OIMVs), cytoplasmic membrane vesicles (CMVs) and tube-shaped membranous structures (TSMSs) [1].

EVs play an essential role in bacterial survival and host interactions due to inter-kingdom signaling and their potential properties in the fecal microbiota–eukaryote interaction [6]. For example, it has been shown that EVs of toxigenic *Bacteroides fragilis* (*B. fragilis*) contribute to bowel disease and colon cancer [7]. EVs of *Akkermansia muciniphila* (*A. muciniphila*) have been shown to play a role in controlling intestinal permeability and regulating intestinal barrier integrity, improving metabolic function and ameliorating obesity in mice [8]. A previous study also demonstrated how *Listeria monocytogenes* (*L. monocytogenes*) produces EVs that carry the majority of listerial virulence proteins, and it uses these EVs for toxin release and mammalian toxicity [9].

Recently, several studies have investigated the secretion of EVs by pure bacterial cultures, while less data are available regarding the secretion of EVs by complex microbial communities or environments. Lagos [10] isolated EVs secreted from fresh pig feces in vitro and observed modifications in their composition and abundance in function under the environmental conditions, especially with respect to carbohydrate availability. Tulkens [2] described the presence of bacterial EVs in human plasma and correlated their abundance with immune activation and barrier integrity in patients with Crohn’s disease (CD), human immunodeficiency viruses (HIVs) and cancer. Further research on the fecal microbiota composition and the derived EVs in feces demonstrated the role of bacterial EVs in the regulation of intestinal immunity and homeostasis and highlighted the protective effect of *A. muciniphila* EVs in the development of dextran-sulfate-sodium-induced colitis [11]. Recently, it was demonstrated how *Staphylococcus aureus* secretes EVs which can be delivered into macrophage cells, stimulating a potent IFN-β response in recipient cells [12]. In addition to feces, the presence of bacterial EVs has also been studied in human breast milk, suggesting a role in the vertical transfer of the fecal microbiota [13]. Furthermore, milk EVs have been demonstrated to be an important source of mRNA and therefore have important potential as a tool for monitoring the clinical stage of bovine leukemia virus infection [14]. However, less data are available in relation to the EVs present in human feces, their metagenomic profiling, their differences according to different diseases associated with an intestinal dysbiosis and their effects on intestinal permeability.

In this study, we first implemented a procedure to isolate fecal-microbe-derived EVs from human feces. To exclude the presence of free DNA in the EV samples, we also investigated the use of a PMA treatment. Second, we compared the fecal microbiota composition with the composition of the fecal-microbe-derived EVs using 16S ribosomal DNA sequencing in healthy subjects and in patients with diarrhea, morbid obesity and CD. Finally, we tested the effect of these EVs on the cellular permeability of Caco-2 cells in vitro.

## 2. Results

### 2.1. PMA Treatment

**Figure 1:**
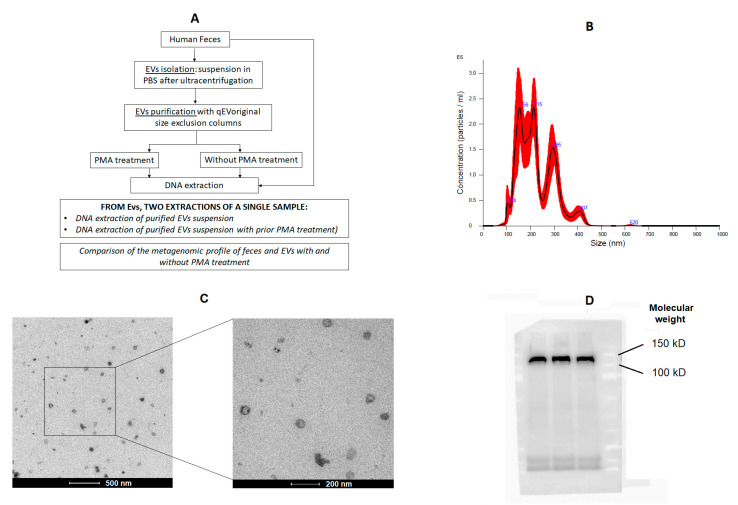
(**A**) Detailed scheme of the assay using Propidium Monoazide (PMA) treatment before DNA extraction and sequencing. Characterization of fecal-microbiota-derived EVs by (**B**) nanoparticle tracking analysis (NTA), (**C**) transmission electron microscopy (TEM) and (**D**) Western blot.

The performance of the purified EVs with qEVoriginal size exclusion columns and PMA treatment was evaluated prior to sequencing and statistical analysis (Figure 1A). Fecal EV purification with qEV IZON columns normally removes free DNA from the samples; however, the objective of this assay was to verify if the final concentration of the EVs (and therefore the DNA concentration) was enough to perform a good-quality sequencing analysis. Therefore, this test allowed us to determine if the purified EVs from feces had too much free DNA, which might have interfered in sequencing results, and if additional PMA treatment was necessary for these samples.

The PMA treatment was applied as previously described, and sequencing and statistical analysis were performed. The α-diversity metrics, including the number of observed genera, Chao1, the reciprocal Simpson index and Simpson evenness, were used to assess community richness and diversity. Good’s coverage was >0.99 for all samples, indicating that although the number of generated sequence reads (on average, 7000) was limited, this sampling effort allowed for the production of an accurate caption of the fecal-microbe-derived EV communities. No significant differences in bacterial richness, diversity or evenness were observed at genus level, regardless of whether the PMA treatment was used or not (Supplementary Figure S1). Regarding the microbiota composition, the purified fecal-microbe-derived EVs presented a few significant differences at genus level compared with those treated with PMA. Post-hoc pairwise differences between the two groups (with and without PMA treatment) were detected only in genus *Alistipes* and *Acidibacter*, which were found to be increased in samples without PMA treatment (Supplementary Figure S2). These results demonstrated that this treatment is not essential for the characterization of human-fecal-microbe-derived EVs when following the protocol implemented in the present study.

### 2.2. Characterization of EVs

After isolation with qEV IZON columns and without PMA treatment, the fecal-microbe-derived EVs showed a typical particle shape and size when analyzed by NTA (Figure 1B) and TEM (Figure 1C). The Western blot analyses revealed the presence a band of bacterial peptidoglycan (Figure 1D).

### 2.3. Microbiome Profile of Fecal-Microbe-Derived EVs and Their Feces of Origin

**Figure 2:**
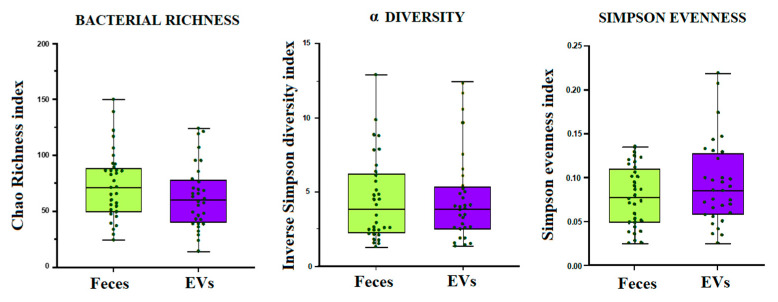
Bacterial diversity for the overall community heterogeneity (Inverse Simpson diversity index), bacterial evenness for the species abundances (Simpson evenness index) and bacterial richness for the total number of species (Chao1 richness index), expressed as a mean value with a standard deviation, for fecal microbiota samples and fecal-microbe-derived EVs when all samples (diarrhea, CD, morbid obesity and controls) were compared together. Statistical differences were calculated according to a non-parametric Wilcoxon matched-pairs signed rank test.

We further compared the microbial profile of the feces from which the EVs were isolated with the microbial profile of their derived EVs. First, a metagenomics analysis of feces was performed to study the microbial profile. The bacterial EVs were purified using qEVoriginal size exclusion columns, and PMA treatment was not performed. Once the EVs were isolated, a metagenomics analysis was performed to sequence and identify the genera from which these EVs originated. The 16S amplicon sequencing yielded 10,000 cleaned reads per sample from which taxonomic identification was obtained. No significant differences were found in bacterial richness (Chao1 richness index), alpha diversity (inverse Simpson index) and evenness (derived from Simpson index) between the feces and fecal-microbe-derived EVs when all samples were compared together (Figure 2).

**Figure 3:**
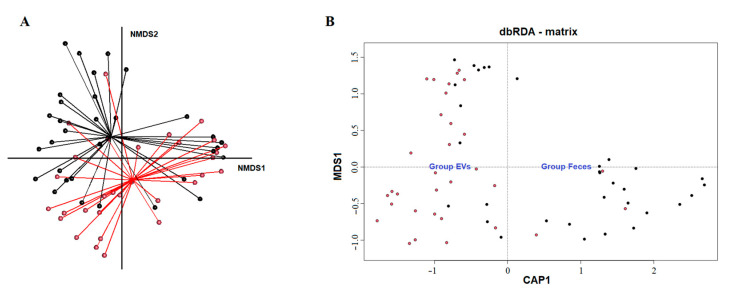
(**A**) Non-metric dimensional scaling model of the microbial profiles illustrating the matrix category of the samples (model with k = 5, stress = 0.094). Samples are represented as dots, with black = fecal microbiota sample and Red = fecal-microbe-derived EVs. Samples are connected to the centromere of each group with lines. (**B**) Distance-based redundancy analysis of the microbial profiles, taking into account the matrix (dbRDA). First dimension, CAP1, is the result of the constrained ordination of the samples based upon the matrix value. Following axes are non-constrained dimensions. Samples are represented as dots. Black= fecal microbiota sample and red = fecal-microbe-derived EVs. Matrix centromeres are represented as group labels in blue.

When the same analysis was performed by group of patients (feces vs. fecal-microbe-derived EVs from the control group and feces vs. fecal-microbe-derived EVs from a group of patients with disease (CD, diarrhea or morbid obesity)), no significant differences were observed between the fecal microbiota and the fecal-microbe-derived EVs after multiple comparisons using the Kruskal–Wallis test with Benjamini–Hochberg FDR corrections (Supplementary Figure S3). AMOVA and HOMOVA analyses showed that the genetic diversity in the fecal-microbe-derived EVs was significantly different from that from fecal bacteria (*p* = 0.037); however, the amount or variation of this genetic diversity in each group (fecal bacteria and EVs) was not significantly different (*p* > 0.05). Finally, the NMDS and dbRDA analyses are shown in Figure 3A and Figure 3B, respectively.

### 2.4. Global Comparison between Feces and Fecal-Microbe-Derived EVs

**Figure 4:**
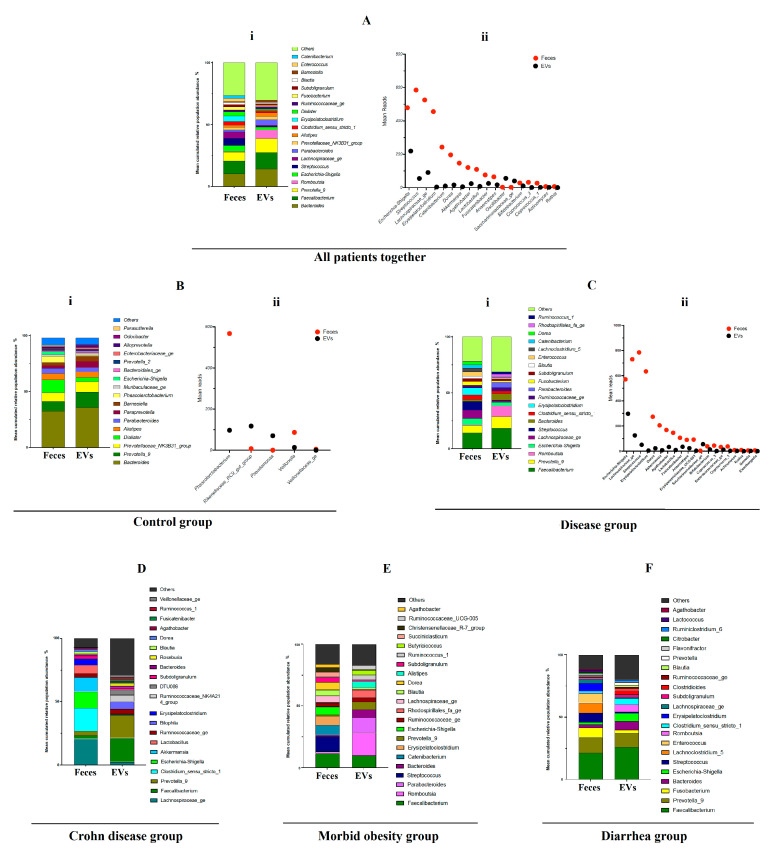
Relative abundance of bacterial genera in fecal microbiota and fecal-microbe-derived EVs. (**A**) All types of subjects (healthy controls and disease patients) were grouped in this analysis. (**B**) Only healthy subjects (n = 9) were considered for this analysis: namely, the control group. (**C**) Only disease patients (n = 23) were considered for this analysis, including diarrheic, morbidly obese and CD patients. (**D**) Only Crohn’s disease subjects (n = 4) were considered for this analysis. (**E**) Only morbid obese subjects (n = 9) were considered for this analysis. (**F**) Only diarrheic subjects (n = 10) were considered for this analysis. (i) Relative abundance of bacterial genera. Only genera with a relative abundance ≥1% were plotted. (ii) Box plot showing bacterial genera statistically different between fecal microbiota and fecal-microbe-derived EVs. Results of DESeq2 with Benjamini–Hochberg FDR corrections.

Twenty-one genera presented a relative abundance greater than 1% in both types of samples: fecal bacteria and EVs (Figure 4A(i)).

Seven dominant genera with a relative abundance >3% in both groups were identified: namely, *Bacteroides*, *Faecalibacterium*, *Prevotella_9*, *Romboutsia*, *Escherichia-Shigella*, *Streptococcus* and *Laschnospiraceae_ge*. Significant differences were observed in 18 different genera (Figure 4A(ii)). Among them, only two taxa, identified as *Oscillibacter* and *Saccharimonadaceae_ge*, were increased in the EV samples, while the remaining 16 genera were significantly increased in the feces samples

### 2.5. Comparison between Feces and Fecal-Microbe-Derived EVs in the Control Group

The comparison between fecal bacteria and EVs obtained only from the control group revealed the presence of 17 genera with a relative abundance greater than 1% and 6 dominant genera with a relative abundance of >3% in fecal bacteria and the EV samples, which were identified as *Bacteroides*, *Prevotella_9*, *Prevotellaceae_NK3B31_group*, *Dialister*, *Alistipes* and *Parabacteroides* (Figure 4B(i)). Few significant differences were observed between the composition of the fecal bacteria and EVs with only five genera implicated, including *Phascolarctobacterium*, *Rikenellaceae_RC9_gut_group*, *Pseudomonas*, *Veillonella* and *Veillonellaceae_ge*. Almost all of these genera were increased in the fecal bacteria and reduced in the EVs with the exception of *Rikenellaceae_RC9_gut_group* and *Pseudomonas*, which were found in higher proportions in the EVs (Figure 4B(ii)). The relative abundance of bacterial genera in the fecal microbiota and fecal-microbe-derived EVs for each patient is shown in Supplementary Figure S4.

### 2.6. Comparison between Feces and Fecal-Microbe-Derived EVs in the Group of Patients with Different Diseases

Finally, we compared the composition of the microbiota from fecal bacteria and EVs in the groups of patients with different diseases (morbid obesity, CD and diarrhea groups together). In the fecal microbiota samples, eleven taxa were identified as dominant, with a relative abundance greater than 3% (Figure 4C(i)), while only five genera were observed in these proportions in the EVs (*Faecalibacterium*, *Prevotella_9*, *Romboutsia*, *Bacteroides* and *Parabacteroides*). Several significant differences between the fecal composition and the composition of the EVs were detected, with 20 different genera implicated (Figure 4C(ii)). Only *Saccharimonadaceae_ge* was found to be increased in EVs, while the remaining 19 genera were all increased in the fecal bacterial samples. The relative abundance of bacterial genera in the fecal microbiota and fecal-microbe-derived EVs per type of disease is shown in Figure 4D (Crohn’s disease), 4E (morbid obesity) and 4F (diarrhea group). The relative abundance of bacterial genera in the fecal microbiota and fecal-microbe-derived EVs for each patient is shown in Supplementary Figure S4.

### 2.7. Comparison between Fecal-Microbe-Derived EVs from Different Diseases

Table 1 shows the significant differences found in the composition of EVs between the four groups of patients. A group of genera was increased (*Bacteroidales* and *Pseudomonas*) or decreased (*Faecalibacterium*, *Ruminococcus*, *Clostridium* and *Subdoligranum*) in EVs from control patients with respect to the rest of the groups. Other genera were also found to be decreased in the control patients with respect to most of the groups (Table 1). There were also some genera that were exclusively increased or decreased, depending on the type of disease, when they were compared to the control group (marked with * in Table 1). Moreover, our findings showed that *Tyzzerella*, *Verrucomicrobiaceae*, *Candidatus_Paracaedibacter* and *Akkermansia* were increased in EVs from the CD group compared to the morbid obesity and diarrhea groups. In addition, *Parabacteroides* was increased in EVs from the morbid obesity group compared to the CD and diarrhea groups. No other significant differences were found.

### 2.8. Intestinal Permeability in Caco-2 Cells

**Figure 5:**
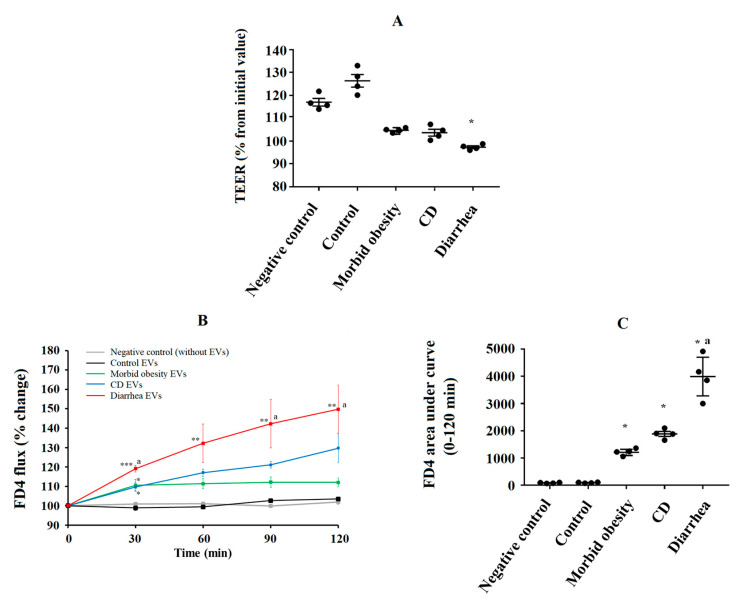
Effect of different types of fecal EVs on the intestinal permeability of Caco-2 cells. Caco-2 cells were plated in 12-well PET Transwell™ inserts of 0.4 μm pore size (Corning Inc., Corning, MA, USA). A suspsension of fecal purified EVs (n = 4) was added for a 24 h incubation period. After, the alteration of the intestinal permeability of Caco-2 cells was assessed by measuring TEER and FD4 unidirectional flux from the apical to basolateral compartments of the Transwell™ at different time points. A negative control was performed using Caco-2 cells without fecal EV treatment. (**A**) TEER was measured using a Millicell^®®^ ERS-2 Voltohmmeter (Merck Millipore, MA, USA) by inserting electrodes into the Transwells. TEER values were measured before and after 24 h of incubation with fecal EVs. Data are presented as percentage of change in TEER value from the initial value (before 24 h of incubation). (**B**) After 24 h of incubation with fecal EVs, Caco-2 cells were washed and treated apically with FD4 (1 mg/mL). The fluorescence in the basolateral chamber was measured before and at different times for 2 h after the addition of FD4. Based on relative fluorescence units, data are presented as percentage of change in FD4 concentrations from the initial value (before 2 h of incubation with FD4). (**C**) Area under curve of the increase in FD4 concentration in the basolateral chamber between before and after 2 h of incubation with FD4. * *p* < 0.05, ** *p* < 0.01, *** *p* < 0.001: significant differences with regard to fecal EVs from control patients. ^a^ *p* < 0.05: significant differences with regard to fecal EVs from patients with morbid obesity. All data are presented as means ± SEM.

We first tested whether fecal EVs induced an alteration of the intestinal permeability of Caco-2 cells by measuring TEER and FD4. Fecal EVs from the different groups of patients were used. We found that Caco-2 cells incubated with fecal EVs from the control patients presented an increase of 29.1 ± 4.0% in the TEER value (Figure 5A).

However, Caco-2 cells incubated with fecal EVs from patients with morbid obesity, CD and diarrhea presented an increase of 12.1 ± 3.52%, 9.9 ± 1.7% and 4.4 ± 1.6%, respectively, in TEER values. The change produced by EVs from patients with diarrhea was significantly lower than the change produced by fecal EVs from the control patients (*p* = 0.0 45). Next, we measured paracellular permeability by monitoring the flux of FD4 through the Transwell. As shown in Figure 5B,C, the fecal EVs from control group did not exert a significant effect on the permeability. Fecal EVs from patients with morbid obesity and CD induced a slightly significant increase in the permeability of Caco-2 cells at 30 min. However, the fecal EVs from patients with diarrhea induced the highest increase at each time in the translocation of FD4 to the basolateral compartment when compared to the control group. Moreover, the increase found with the diarrhea fecal EVs was also significantly higher than with the fecal EVs from patients with morbid obesity. Therefore, the permeability of the Caco-2 cells was modified by fecal EVs according to their origin.

## 3. Discussion

Sequencing methods do not discriminate between live (dormant cells and non-growing or growing cells, which are metabolically active) or dead bacteria. In our study, the analysis of the PMA-treated EVs did not present enough differences in richness, alpha diversity or Good’s coverage to be statistically different from those that were not treated with PMA. This method is recognized as a valuable tool for the distinction of dead/viable cells since PMA treatment is a DNA-intercalating agent that acts on free DNA and penetrates cells with compromised membranes [14]. Regarding the composition of EVs, significant differences were only found for two genera, indicating that most populations can be found in the same proportions in EVs treated and not treated with PMA. Therefore, this treatment is not essential for the metagenomics analysis of fecal-microbe-derived EVs from human feces when the protocol implemented in the present study is followed. The use of qEV original size exclusion columns for the purification of fecal EVs appears to be sufficiently efficient to remove free DNA from the fecal EVs.

Microbe-derived EVs have been directly associated with disease development [15]. However, there are few studies on the composition of fecal-microbe-derived EVs compared with their feces of origin, and a large proportion of these studies focused on colorectal cancer and inflammatory bowel disease (IBD) patients [16,17,18]. In our study, the overall bacterial richness and diversity were not significantly different between the two types of samples studied (fecal bacteria and fecal-microbe-derived EVs) within the control group or within patients with disease. However, differences were detected in the microbial composition of EVs in relation to the fecal microbiota. In general, higher proportions of various genera were found in the fecal microbiota compared with the EVs. Previous studies have also demonstrated that the protein composition of fecal EVs differs from that of fecal samples in IBD patients [16]. These findings may suggest that the different bacterial genera secrete variable proportions of EVs which, in turn, may be influenced by the patient’s intestinal disease. These fecal-microbe-derived EVs may be used as novel biomarkers to detect various intestinal diseases, as proposed by Park for colorectal cancer [15]. We also observed more differences in the microbiota structure between the fecal bacteria and EVs in patients with disease than in the control group. However, the main limitation of this study is the low number of recruited patients in each group, which did not allow us to describe the fecal-microbiota-derived EVs that are candidates for predicting each disease. Nevertheless, our results provide preliminary data to further study how the composition of these fecal-microbe-derived EVs is modified in different diseases and to analyze whether these EVs may be involved in the microbiota–host interaction. Previously, significant compositional differences were demonstrated in obese and diabetic rats compared to normal rats in terms of the composition of microbial EVs [19]. Another study also showed that the composition of intestinal EVs was greatly altered after vertical sleeve gastrectomy in mice [20]. As bacteria proliferate, the secretion of EVs should increase in line with the increase in the relative abundance of taxa [10]. However, it is possible that bacteria, depending on the group to which they belong, are capable of producing a greater or lesser number of EVs. Furthermore, this production may be influenced by the presence of other bacterial communities and by the physiological conditions of the environment. This could be a hypothesis to explain the increase in EVs in certain bacterial groups with respect to their percentage in fecal microbiota.

In this study, the control patients presented high proportions of *Pseudomonas* and *Rikenellaceae_RC9_gut_group* in fecal-microbe-derived EVs but lower proportions of *Phascolarctobacterium*, *Veillonella* and *Veillonellaceae_ge* when compared with the fecal bacterial samples. The *Pseudomonas* genus, specifically *Pseudomonas fragi*, also commonly produced important levels of EVs during growth. These vesicles display considerable proteolytic activity but are not associated with bacteriocinogenicity. They most likely act in the physiological distribution of extracellular proteinases [21]. Regarding *Rikenellaceae_RC9_gut_group*, only one previous study described an increase in their EVs in patients with colorectal cancer [15]. In addition, a high proportion was found after fecal microbiota transplantation upon *Salmonella Enteritidis* infection in chicks [22]. Moreover, the supplementation with probiotics in broilers had a promoting effect on the growth performance and increased the colonization of beneficial bacteria in the cecum as *Rikenellaceae_RC9_gut_group* [23]. It is involved in degrading carbohydrates [24] and metabolizes lipids [25]. In addition, members of the *Veillonellaceae* family are often found in association with gut inflammation [26] and are more abundant in patients with IBD, fibrosis and other diseases [27,28]. Taken together, these data seem to suggest that the EVs derived from certain bacteria might have an important role in the maintenance of intestinal homeostasis.

In our disease patients, we observed differences between the composition of the fecal bacteria and EVs in a total of 20 genera, all of which showed decreased proportions in EVs except *Saccharimonadaceae_ge*, which was increased in the EVs. In the literature, there is no specific information about the presence of *Saccharimonadacea*-derived EVs in the feces of patient; therefore, its role in the gut requires further investigation. EVs belonging to other genera that were found to be decreased in our study have been previously studied due to their possible effects on intestinal diseases. An example of this includes *Akkermansia* (*A. muciniphila*)-derived EVs, which have been reported to act as a functional moiety for controlling gut permeability and regulating the intestinal barrier integrity in mice [8]. Other important bacterial groups that showed significant differences between the composition of the fecal microbiota and EVs are *Lactobacillus* and *Bifidobacterium*. The EVs of *Lactobacillus plantarum Q7* have been demonstrated to alleviate induced colitis symptoms and histological damage in mice. They also reduced the levels of proinflammatory bacteria *(Proteobacteria*) and increased the levels of anti-inflammatory groups (*Bifidobacterium* and *Muribaculaceae*) [29]. The EVs of *Bifidobacterium longum* can export several cytoplasmic proteins that could be involved in bifidobacterial adhesion and survival in the gastrointestinal tract [30].

Our in vitro experiment demonstrated that fecal EVs act as regulators of epithelial barrier integrity with differences depending on the disease of the patients. This is in accordance with the different compositions of fecal-microbe-derived EVs that are dependent on the type of disease. In contrast to most studies, our findings describe the effects of EVs from a mixture of fecal bacteria, not from a specific bacterium. Several studies with different species of fecal microbiota have demonstrated the role of bacterial-derived EVs as modulators of epithelial barrier integrity [31]. In this context, the fecal microbiota-derived EVs, besides the host-derived EVs [31], could be involved in the regulation of gut homeostasis by enhancing the intestinal permeability, a condition that subsequently leads to inflammatory and metabolic diseases [32]. This increased gut permeability would allow for the passage of endotoxins and luminal antigens into the intestinal lamina propria, initiating a mucosal immune response that causes chronic, low-grade inflammation, prompting metabolic disorders such as insulin resistance and obesity [31]. Possible differences in the surface cargo molecules, such as microbe-associated molecular patterns (MAMPs), could be mediating the adhesion of these fecal-microbiota-derived EVs to host epithelial cells and, consequently, the downstream effects [33]. Moreover, in a later study, it would be interesting to analyze the metabolic and transcriptomic changes produced by these fecal EVs in different types of cells.

A limitation of this study was that we did not characterize the total composition of these EVs, i.e., we did not analyze their protein, RNA, DNA and lipid contents. These factors could be associated with the effects produced by these EVs. In this study, we only focused on analyzing the genera from which the EVs originated by metagenomic analysis. In addition, although the method used in this study to isolate the EVs has been previously described and used [34,35,36], it is possible that it could be improved by performing EV isolation prior to freezing in order to minimize the presence of intracellular artifacts/contaminants from microorganisms/cells derived from the freezing process. This point will require further study to analyze the differences between these two methodologies.

In summary, we conducted a metagenomic study to reveal associations between the fecal microbiota and the microbial composition of EVs in control subjects and in patients with disease. We found that fecal-microbiota-derived EVs from control subjects have a metagenomic profile closely similar to that of the fecal microbiota. However, we have shown that the presence of a dysbiotic fecal microbiota in different diseases is accompanied by an altered composition of fecal-microbe-derived EVs. Therefore, our findings demonstrate that diseases such as diarrhea, CD or morbid obesity alter the microbial composition of EVs in relation to the fecal microbiota. On the other hand, we found an increase in intestinal permeability with fecal EVs from patients with different diseases. We suggest that the fecal-microbiota-derived EVs from certain bacteria might cause increased intestinal permeability as part of their infectious mechanisms, while other bacterial strains attenuate inflammation and reinforce the gut barrier integrity [37]. We postulated the importance of controlling the balance between the different subsets of fecal microbiota and their EVs in the development of diseases associated with altered intestinal permeability. However, the cause-and-effect relationships and the role of these fecal-microbiota-derived EVs, as mediators of interspecies interactions and as novel biomarkers, in the course of a disease require future careful, experimental studies.

## 4. Materials and Methods

### 4.1. Patient Recruitment

Our cohort study included 32 patients: 9 healthy volunteers, 10 diarrheic patients, 9 patients with morbid obesity and 4 patients with CD. These diseases were chosen because they demonstrates a clear alteration of fecal microbiota [38,39,40]. In those patients with diarrhea, neither parasites nor Cryptosporidium were isolated in the feces, they had normal flora, no *Salmonella*, *Shigella*, *Campylobacter*, *Yersinia* and *Aeromonas* were isolated, and the presence of *Clostridium difficille* toxin and adenovirus and rotavirus antigens was negative. Fecal samples were collected from all patients (n = 32) and immediately stored at −80 °C in the Virgen de la Victoria University Hospital Biobank (Andalusian Public Health System BioBank) until analysis. All participants were of Caucasian origin. All participants gave their written informed consent, and the study protocol was carried out in accordance with the ethical guidelines of the Declaration of Helsinki. The study was approved by the Malaga Provincial Research Ethics Committee, Malaga, Spain (PI18/01652, PE-0098-2019).

### 4.2. Isolation of EVs from Human Feces

A total of 10 g of feces was inoculated into 40 mL of sterile, phosphate-buffered saline (PBS) and homogenized. The EVs were then isolated through centrifugation as previously described with some modifications [10]. Briefly, a first centrifugation of the homogenate was performed (40 min, 4000× *g*, and 4 °C. The supernatant was recovered and filtered using sterilized vacuum filtration units, Rapid-Flow™ filters MF 75, 1000 mL of Nalgene^®^ and 0.2 μm of cold ice (Thermo Fisher Scientific, Waltham, MA, USA). The filtrate was transferred to 10 mL polycarbonate, open-top, thick-wall tubes and ultracentrifuged at 100,000× *g* for 3 h at 4 °C with a fixed-angle rotor (Type 70.1 Ti) in a Beckman Optima XL-100K ultracentrifuge (Beckman Coulter Life Sciences, Indianapolis, IN, USA). Pellets were resuspended in 200 µL of PBS and the EVs were purified using qEVoriginal size exclusion columns of 70 nnm (Izon Science Europe Ltd., Oxford, UK), following the manufacturer’s recommendations. Fractions 6–8 (enriched in EVs) were collected, mixed, concentrated with Vivaspin^®^ 6 100K centrifugal concentrators (Sartorius AG, Göttingen, Germany), aliquoted and frozen at −80 °C until use. This protocol was used to separate bacteria and other contaminating soluble molecules, such as toxins and proteins, from the EVs. These aliquots of fecal EVs were used for treatment with propidium monoazide, metagenomic analysis, transmission electron microscopy, nanoparticle tracking analysis, Western blot and for the incubation of Caco-2 cells.

### 4.3. Transmission Electron Microscopy (TEM) of EVs

The isolated EVs (n = 4; one from a healthy control, one from a CD patient, one from a diarrheic patient and one from a patient with morbid obesity) were fixed in 2% paraformaldehyde—0.1 M PBS for 30 min. A glow discharge technique (60 s, 7.2 V, using a Bal-Tec MED 020 Coating System) was applied over carbon-coated copper grids, and these grids were immediately placed on top of sample drops for 15 min. Then, the grids with adherent EVs were washed in a 0.1 M PBS drop. Additional fixation in 1% glutaraldehyde was performed for 5 min. After washing the grids properly in distilled water, the grids were contrasted with 1% uranyl acetate and embedded in methylcellulose. Excess fluid was removed and allowed to dry before examination with a transmission electron microscope FEI Tecnai G2 Spirit (ThermoFisher Scientific, Waltham, MA, USA). All images were acquired using a Morada digital camera (Olympus Soft Image Solutions GmbH, Münster, Germany). The magnification used for the TEM images was 49,000×.

### 4.4. Nanoparticle Tracking Analysis (NTA)

The EV size and concentration were assessed using the NanoSight NS300 system (Malvern Panalytical, Malvern, UK) (n = 3; one from a morbidly obese patient, one from a diarrheic patient and one from a healthy control). Particles were automatically tracked and sized-based on Brownian motion and the diffusion coefficient. The EVs were resuspended and diluted with 0.22 μm filtered PBS at a concentration range 109 particles/mL, and 1 mL was used for NanoSight analysis. Five replicates of 30 s videos were captured to analyze the concentration and size distribution of the EVs at the detection threshold of 5. A data analysis was performed using NanoSight analysis software.

### 4.5. Western Blot of EVs

Fecal EVs (n = 3; one from a CD patient, one from a morbidly obese patient and one from a healthy control) were lysed with 1× RIPA buffer (Thermo Fisher (Kandel) GmbH, Kandel, Germany) and supplemented with a protease inhibitor cocktail (Merck KGaA, Darmstadt, Germany). The protein lysate was incubated with the same volume of Laemmli Buffer 2× (Bio-Rad Laboratories, Inc., Hercules, CA, USA) and supplemented with 2-mercaptoethanol (5%) at 95 °C for 5 min. The samples were subjected to 4–20% SDS-PAGE (NB12-420) (NuSep, Inc., Germantown, MD, USA) and transferred onto polyvinylidene fluoride membranes (Trans-Blot Turbo Midi 0.2 µm PVDF Transfer Packs) (Bio-Rad Laboratories, Inc., Hercules, CA, USA) at 13 V and 1.1 A for 20 min. The membranes were subsequently blocked in PBS–bovine serum albumin (BSA) 5% for 1 h at room temperature. The membranes were then incubated for 48 h at 4 °C with a mouse monoclonal anti-bacterial peptidoglycan antibody, clone 3F6B3 (Merck KGaA, Darmstadt, Germany). This antibody is specific to the three-dimensional polymer complex structure of bacterial peptidoglycan. The membranes were washed three times with 0.05% Tween-20 washing buffer in PBS and incubated with a horseradish-peroxidase-conjugated secondary antibody (VeriBlot for IP Detection Reagent (HRP), ab131366) (Abcam, Cambridge, UK) for 3 h at room temperature. Finally, after another three washes, the membranes were revealed with Clarity Western ECL substrate (Bio-Rad Laboratories, Inc., Hercules, CA, USA). The proteins were visualized by an ImageQuant LAS 4000 (GE Healthcare, Buckinghamshire, UK).

### 4.6. Propidium Monoazide (PMA) Treatment

The procedure used to isolate the microbe-derived EVs could result in the presence of a small percentage of free DNA in the sample. Therefore, we tested sample treatment with PMA [41] in order to detect the co-extraction and amplification of the nonprotected DNA of the membrane-compromised EVs. Furthermore, we wanted to evaluate the optimal separation of EVs from free DNA using qEVoriginal size exclusion columns of 70 nnm (Izon Science Europe Ltd., Oxford, UK). For this assay, seven samples from patients were evaluated (two from healthy volunteers, two from diarrheic patients, two from morbidly obese patients and 1 from a patient with CD). In total, 100 µL of each sample was centrifuged at 5000× *g* in duplicate from which one was left untreated and the other one was treated with PMA (PMAxx™ dye) (Biotium, Fremont, CA, USA) prior to DNA extraction (Figure 1A). The manufacturer’s protocol for PMA treatment was used and involved the use of the PMA-Lite™ LED Photolysis Device (Biotium, Fremont, CA, USA). The statistical analyses were performed with the seven samples tested.

### 4.7. DNA Extraction

The total DNA was extracted from the EVs that were treated and not treated with PMA (Izon Science Europe Ltd., Oxford, UK) and directly from the fecal samples using DNeasy blood and Tissue Kits (QIAGEN Science, Hilden, Germany), following the manufacturer’s recommendations. Briefly, after the isolation and purification of the EVs from feces, DNA extraction was performed. Once this DNA was obtained, seven of these DNA samples from the EVs were aliquoted in duplicate to treat one half with PMA. In parallel, a DNA extraction was also performed in all fecal samples used for the isolation of EVs. The DNA was eluted into DNase/RNase-free water and its concentration and purity were evaluated using a NanoDrop ND-1000 spectrophotometer (NanoDrop Technologies, Inc., Wilmington, DE, USA). Extracts were stored at −20 °C until use.

### 4.8. Libraries Preparation and Sequencing

Libraries and sequencing were performed as previously described [42]. Briefly, amplification of the V1-V3 regions of the 16S rRNA bacterial gene was performed using the primers 5′-GAGAGTTTGATYMTGGCTCAG-3′ forward and 5′-ACCGCGGCTGCTGGCAC-3′ reverse with overhand adapters. Amplicons were purified using Agencourt AMPure XP bead kit (Beckman Coulter, Pasadena, CA, USA), indexed using Nextera XT index primers 1 and 2 (Illumina, San Diego, CA, USA), quantified by Quant-IT PicoGreen (Thermo Fisher Scientific, Waltham, MA, USA) and diluted to a concentration of 10 ng/μL. DNA samples were quantified by qPCR with a KAPA 170 SYBR^®®^ FAST qPCR Kit (Kapa Biosystems, Wilmington, MA, USA). Samples were normalized, pooled and sequenced using Illumina MiSeq technology with v3 reagents (Illumina, San Diego, CA, USA), using paired end reads by GIGA Genomics platform (Liège, Belgium). A bacterial community composed of known proportions of *Carnobacterium maltaromaticum*, *Lactococcus lactis* subsp. *cremoris*, *Leuconostoc carnosum*, *Pseudomonas aeruginosa* and *Streptococccus thermophilus* was used as a positive control. Negative controls were used in their entirety for DNA extraction, library preparation and sequencing.

### 4.9. Bioinformatics, Ordination and Statistical Analysis

Sequence reads were processed using Mothur v1.44.3 and VSearch for alignment, clustering and chimera detection, respectively [43,44]. The sequences were clustered into operational taxonomic units (OTUs) at an identity of 97%. The SILVA 138 database of full-length 16S rDNA gene sequences was used for the alignments of unique sequences and taxonomical assignations. For each sample, a subsampling dataset containing 10,000 representative, cleaned reads was retained (mean: 10,000, SD: 0) and used to generate OTUS (cut off: 0.03) as well as to evaluate several ecological indicators.

All statistical analyses were performed at the genus level. Regarding alpha diversity (reciprocal Simpson diversity index and Simpson evenness), Goods’s coverage and population richness (Chao1 estimator of richness) were calculated using Mothur v1.44.3 and compared between two groups using a Wilcoxon matched-pairs signed rank test (PRISM 8) (GraphPad Software, Boston, MA, USA) or between three or more groups using Kruskal–Wallis multiple testing with Benjamini–Hochberg FDR corrections (PRISM 8) (GraphPad Software, Boston, MA, USA). Bar plots were built using PRISM 8, including only genera with a relative abundance >1%. The β-diversity was estimated with the Bray–Curtis dissimilarity index using Mothur (v1.44.3) and R for graphical analysis (v1.2.5033). Non-metric multidimensional scaling (NMDS) was performed using Mothur and was considered satisfying when the stress value was <0.20. An AMOVA (analysis of molecular variance) and a HOMOVA (homogeneity of molecular variance) were performed using Mothur in order to reveal eventual significant population structure differences and to determine if the genetic diversity within two or more populations was homogeneous [44]. A distance-based redundancy analysis (dbRDA) was constructed using RStudio. Post-hoc pairwise differences between groups were assessed with Deseq2 package in R, and differences were then identified with Kruskal–Wallis tests using Benjamini–Hochberg FDR correction [45].

### 4.10. In Vitro Cell Culture

Caco-2 (ECACC, Cat. No. 09042001) epithelial cell lines were maintained in complete medium (Dulbecco’s modified Eagle’s Medium (DMEM) of high glucose with L-glutamine (Biowest, Nuaillé, France) supplemented with 10% heat-inactivated fetal bovine serum (FBS) (Biowest, Nuaillé, France), 1% penicillin/streptomycin (Biowest, Nuaillé, France) and 1% MEM non-essential amino acids (Sigma-Aldrich, St. Louis, MO, USA) under standard conditions inside a humidified cell culture incubator at 37 °C with 5% CO_2_. Caco-2 cells were harvested by washing three times in sterile DPBS, followed by treatment with trypsin-EDTA. Harvested cells were counted and seeded in 12-well PET Transwell™ inserts of 0.4 μm pore size (Corning Inc., Corning, MA, USA) at 10^5^ cells/insert by adding 0.5 mL of cell suspension. The apical and basal cell culture media, 0.5 mL and 1.5 mL respectively, were changed every two days. Cells were maintained for approximately 3 weeks in the same medium to allow for full cell differentiation. The culture medium was changed, and 1 μg of protein from the purified EVs suspension was added for 24 h of incubation [8]. The protein concentration of the purified fecal EV suspension was determined using the bicinchoninic acid (BCA) assay (Thermo Fisher Scientific, Waltham, MA, USA). After 24 h of incubation with fecal EVs (n = 4 for each group of patients), the trans-epithelial electrical resistance (TEER) and para-cellular permeability were measured to analyze the EV-induced changes.

### 4.11. Trans-Epithelial Electrical Resistance

TEER was measured using a Millicell^®®^ ERS-2 Voltohmmeter (Merck Millipore, Burlington, MA, USA). Once Caco-2 cells reached a TEER > 1000 (Ω·cm^2^), experiments with the purified fecal EV suspension were performed as described above. TEER values were obtained by subtracting cell-free filter readings and correcting for the surface area (1.1 cm^2^). All readings of TEER were repeated across triplicate sample Transwells. TEER values were expressed as the percentage of change with respect to the TEER value obtained prior to the incubation with purified fecal EV suspension. Data were presented as means ± SEM (n = 4).

### 4.12. Paracellular Permeability

The Caco-2 monolayer paracellular permeability was assessed by measuring the unidirectional flux of fluorescein isothiocyanate (FITC)-dextran (FD4; 4000 Da, Sigma-Aldrich, Saint-Louis, MO, USA) from the apical to the basolateral compartments of the Transwell™. The complete DMEM medium was removed from the apical and basolateral compartments, replaced with Krebs Ringer Bicarbonate Buffer Hepes Albumin (KRBHA), and equilibrated for 1 h at pH 7.4. The KRBHA medium was replaced again, and 25 mg/mL stock solution of FD4 was added to the apical compartment at time zero to obtain a final concentration of 1 mg/mL. An aliquot of 100 μL from the basolateral compartment was removed every 30 min over 2 h, followed by replacement with fresh KRBHA. Samples were transferred onto Nunclon^®®^ MicroWell plates (Thermo Scientific, MA, USA) and the fluorescence of FD4 was measured in a microplate fluorescence reader (FLx 800, Bio-tek Instruments Inc., Winooski, VT, USA) with an excitation of 485/20 nm and an emission of 528/20 nm. A negative control was performed with the Caco-2 cells without fecal EV treatment. A positive control was performed with the Caco-2 cells and 5 mM EGTA instead of fecal EVs. EGTA causes a breakdown of the tight junctions by sequestering bivalent ions independently of inflammatory stimuli [46]. Based on the relative fluorescence units, FD4 concentrations were expressed as the percentage of change from Caco-2 cells without fecal EV treatment. All the results were analyzed in triplicate. Data were presented as means ± SEM (n = 4).

### 4.13. Statistical Analysis

All data were analyzed with GraphPad Software (Prism 8.1.1) (GraphPad Software, San Diego, CA, USA). Differences between groups were compared using Kruskal–Wallis tests followed by post hoc analyses using Dunn’s test. Values were considered to be statistically significant when *p* < 0.05.

## Figures and Tables

**Table 1:** Comparisons between the composition of fecal-microbe-derived EVs from different diseases. Only those genera with significant differences are shown (*p* < 0.05). The percentage of each bacterial genus is shown in parentheses. ^†^ Disease groups included morbid obesity, Crohn’s disease (CD) and diarrhea groups. * *Genus*: genera that were exclusively increased or decreased in that disease when they were compared to the control group, and that were not significant in other diseases compared to the control group.

	Genera Decreased in Fecal-Microbe-Derived EVs from Control Patients			Genera Increased in Fecal-Microbe-Derived EVs from Control Patients		
	Bacteria Genus	Percentage in Control	Percentage in Disease	Bacteria Genus	Percentage in Control	Percentage in Disease
Control vs. Disease ^†^	*Faecalibacterium*	<0.01%	13.4%	*Bacteroidales*	1%	0.01%
	*Romboutsia*	<0.01%	4.9%	*Pseudomonas*	0.3%	<0.01%
	*Ruminococcus*	0.01%	1.8%			
	*Clostridium*	<0.01%	2.9%			
	*Butyricicoccus*	<0.01%	0.7%			
	*Subdoligranum*	<0.01%	1.6%			
	*Blautia*	<0.01%	1.3%			
	*Lachnospiraceae*	0.02%	3.4%			
	*Oscillibacter*	<0.01%	0.3%			
	*Roseburia*	<0.01%	0.4%			
	*Saccharimonadaceae*	<0.01%	0.2%			
	*Cutibacterium*	<0.01%	0.2%			
	*Christensenellaceae*	<0.01%	0.02%			
	*Ruminiclostridium*	<0.01%	0.2%			
	*Intestinibacter*	<0.01%	0.2%			
	*Lachnoclostridium*	<0.01%	0.4%			
	*Agathobacter*	<0.01%	0.7%			
Control vs. Morbid Obesity	*Faecalibacterium*	<0.01%	7.8%	*Enterobacteriaceae*	0.9%	0.1%
	*Romboutsia*	<0.01%	9.2%	*Bacteroidales*	1%	0.01%
	*Ruminococcus*	0.1%	2.4%	*Pseudomonas*	0.3%	<0.01%
	*Clostridium*	<0.01%	0.5%	*Dialister*	6.5%	0.1%
	*Subdoligranum*	<0.01%	1.9%	*Rikenellaceae*	0.5%	<0.01%
	*Lachnospiraceae*	0.2%	2.3%			
	*Christensenellaceae*	<0.01%	1.2%			
	*Oscillibacter*	<0.01%	0.1%			
	*Saccharimonadaceae*	<0.01%	0.2%			
	*Cutibacterium*	<0.01%	0.1%			
	*Ruminiclostridium*	0.01%	0.6%			
	* Intestinibacter	<0.01%	0.4%			
	* Megamonas	<0.01%	0.2%			
Control vs. CD	*Faecalibacterium*	<0.01%	7.6%	*Bacteroidales*	1%	<0.01%
	*Ruminococcus*	<0.01%	0.7%	*Enterobacteriaceae*	0.8%	0.2%
	*Clostridium*	<0.01%	9%	*Pseudomonas*	0.3%	<0.01%
	*Butyricicoccus*	<0.01%	0.2%	*Parabacteroides*	3.5%	0.02%
	*Subdoligranum*	<0.01%	1.5%	*Muribaculaceae*	1.4%	<0.01%
	*Blautia*	<0.01%	0.8%	*Butyricimonas*	0.6%	<0.01%
	*Lachnospira*	<0.01%	0.3%	*Prevotella*	0.9%	<0.01%
	*Christensenellaceae*	<0.01%	0.4%			
	*Roseburia*	<0.01%	0.9%			
	* Fusicatenibacter	<0.01%	0.7%			
	* Agathobacter	<0.01%	0.7%			
	* Veillonellaceae	<0.01%	0.5%			
	* Tyzzerella	<0.01%	0.1%			
Control vs. Diarrhea	*Faecalibacterium*	<0.01%	20.8%	*Parabacteroides*	3.48%	0.2%
	*Romboutsia*	<0.01%	2.9%	*Dialister*	6.5%	0.11%
	*Ruminococcus*	<0.01%	0.4%	*Muribaculaceae*	1.4%	0.02%
	*Clostridium*	<0.01%	2.5%	*Odoribacter*	0.8%	0.01%
	*Butyricicoccus*	<0.01%	0.3%	*Bacteroidales*	1%	0.01%
	*Subdoligranum*	<0.01%	1.4%	*Pseudomonas*	0.3%	0.01%
	*Blautia*	<0.01%	0.8%	*Rikenellaceae*	0.5%	<0.01%
	*Oscillibacter*	<0.01%	0.4%	*Butyricimonas*	0.6%	<0.01%
	*Roseburia*	<0.01%	0.3%	*Prevotellaceae*	0.5%	<0.01%
	*Saccharimonadaceae*	<0.01%	0.3%	* Alistipes	7%	0.01%
	*Cutibacterium*	<0.01%	0.3%	* Barnesiellla	3%	0.02%
	*Ruminiclostridium*	<0.01%	0.5%	* Paraprevotella	3%	<0.01%
	* Peptoclostridiaceae	<0.01%	1%	* Coprobacter	0.3%	<0.01%
				* Mollicutes	0.06%	<0.01%
	**Genera Decreased in Fecal-Microbe-Derived EVs from Control Patients**			**Genera Increased in Fecal-Microbe-Derived EVs from Control Patients**		
	**Bacteria Genus**	**Percentage** **in Control**	**Bacteria Genus**	**Percentage** **in Control**	**Bacteria Genus**	**Percentage** **in Control**
Morbid obesity vs. CD	*Tyzzerella*	<0.01%	0.1%	*Parabacteroides*	5.2%	0.02%
	*Verrucomicrobiaceae*	<0.01%	0.3%			
	*Candidatus_Paracaedibacter*	<0.01%	0.3%			
	*Akkermansia*	0.05%	4.3%			
Morbid obesity vs. Diarrhea				*Parabacteroides*	5.2%	0.2%
	**Genera Decreased in Fecal-Microbe-Derived EVs from Control Patients**			**Genera Increased in Fecal-Microbe-Derived EVs from Control Patients**		
	**Bacteria Genus**	**Percentage** **in Control**	**Bacteria Genus**	**Percentage** **in Control**	**Bacteria Genus**	**Percentage** **in Control**
Diarrhea vs. CD	*Ruminococcaceae*	<0.01%	1.4%	*Romboutsia*	3%	0.02%
	*Tyzzerella*	0.01%	0.1%	*Prevotella*	0.6%	0.01%
	*Candidatus_Paracaedibacter*	<0.01%	0.3%			
	*Akkermansia*	<0.01%	4.3%			

## Data Availability

The original libraries are publicly available under the BioProject ID PRJNA875626 at the National Center for Biotechnology Information (NCBI) (https://www.ncbi.nlm.nih.gov/bioproject/?term=PRJNA875626) (date: 1 September 2022). The data that support the findings of this study are available from the corresponding author, [E.G.F.], upon reasonable request.

## Supplementary Materials

The supporting information can be downloaded at: https://www.mdpi.com/article/10.3390/ijms24054971/s1.
