# Supplementary figures and images for: The Metagenomic Composition and Effects of Fecal-Microbe-Derived Extracellular Vesicles on Intestinal Permeability Depend on the Patient’s Disease
Source: Int J Mol Sci. 2023 Mar 4;24(5):4971. doi: 10.3390/ijms24054971 (PMC10002483; doi:10.3390/ijms24054971)

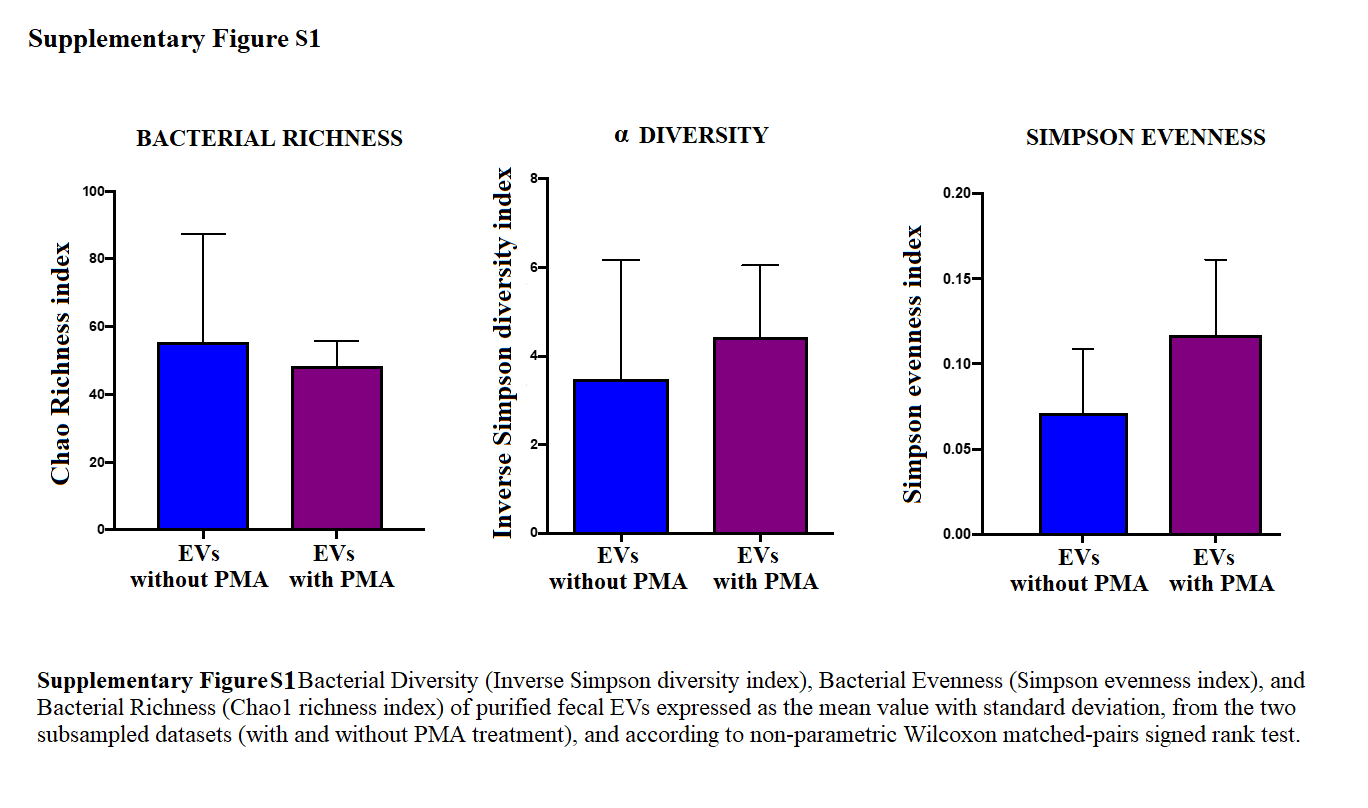

Supplement: Supplementary file 1 [file ijms-24-04971-s001.zip › Supplementary Figure S1.tif]

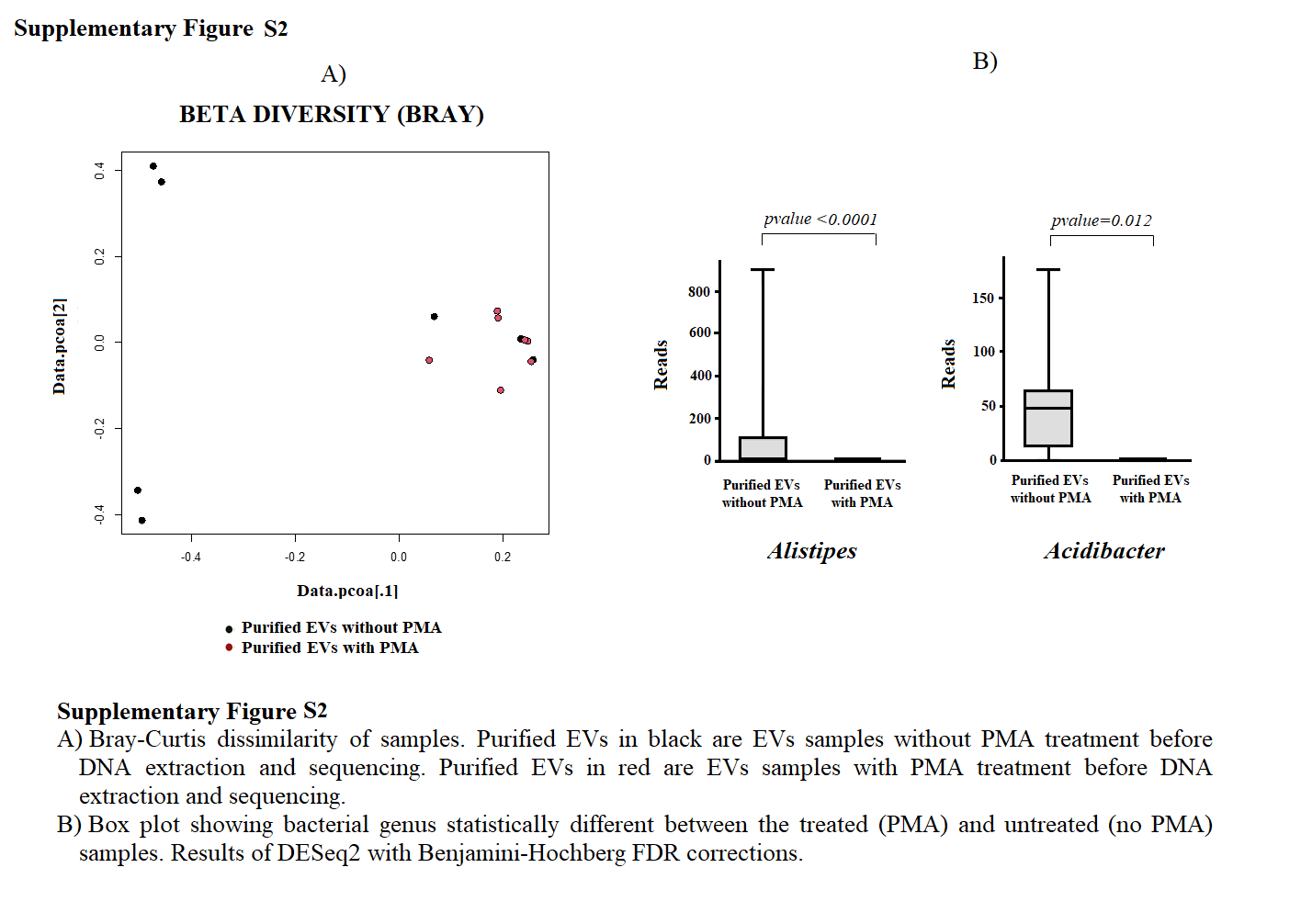

Supplement: Supplementary file 1 [file ijms-24-04971-s001.zip › Supplementary Figure S2.tif]

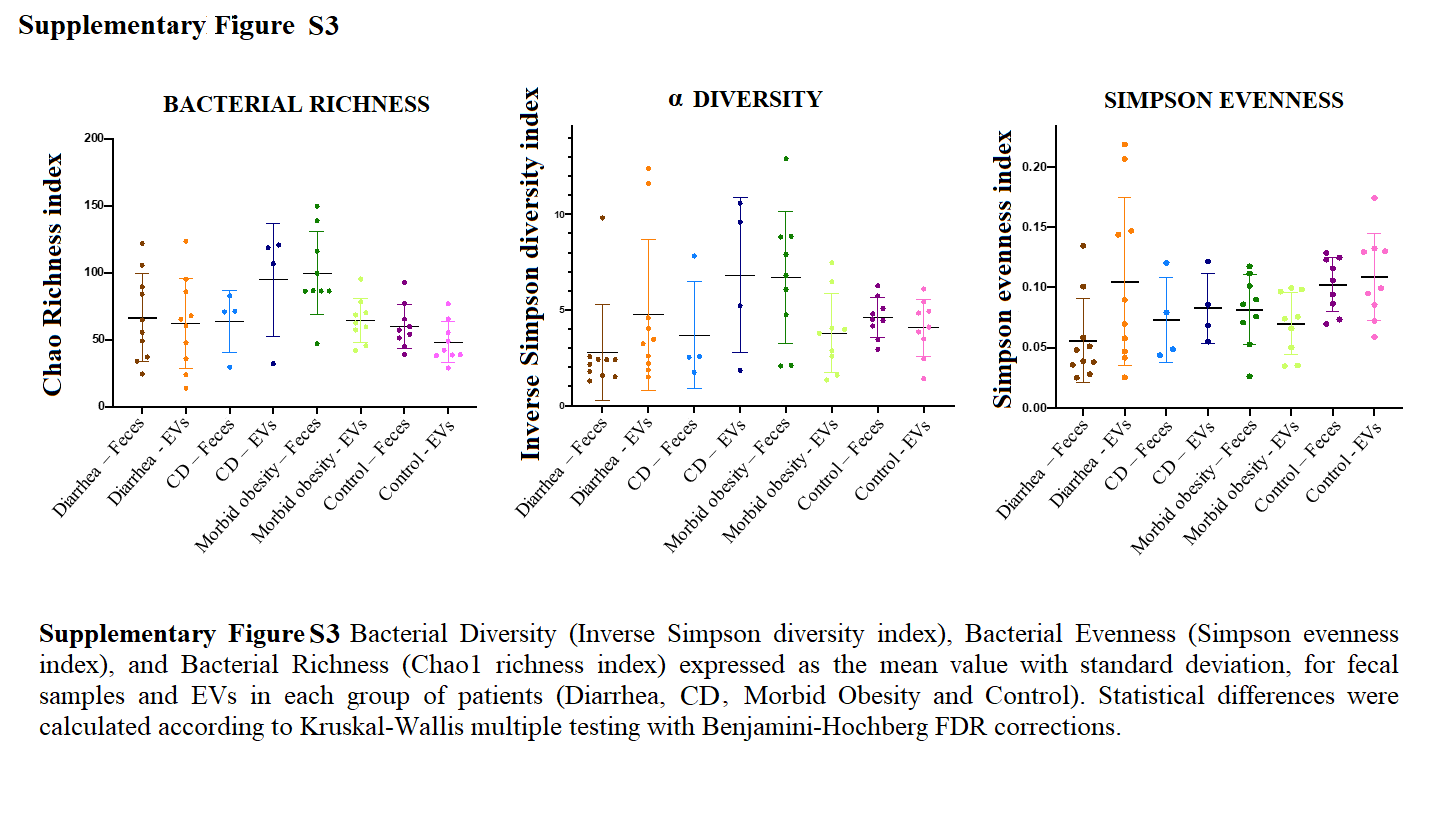

Supplement: Supplementary file 1 [file ijms-24-04971-s001.zip › Supplementary Figure S3.tif]

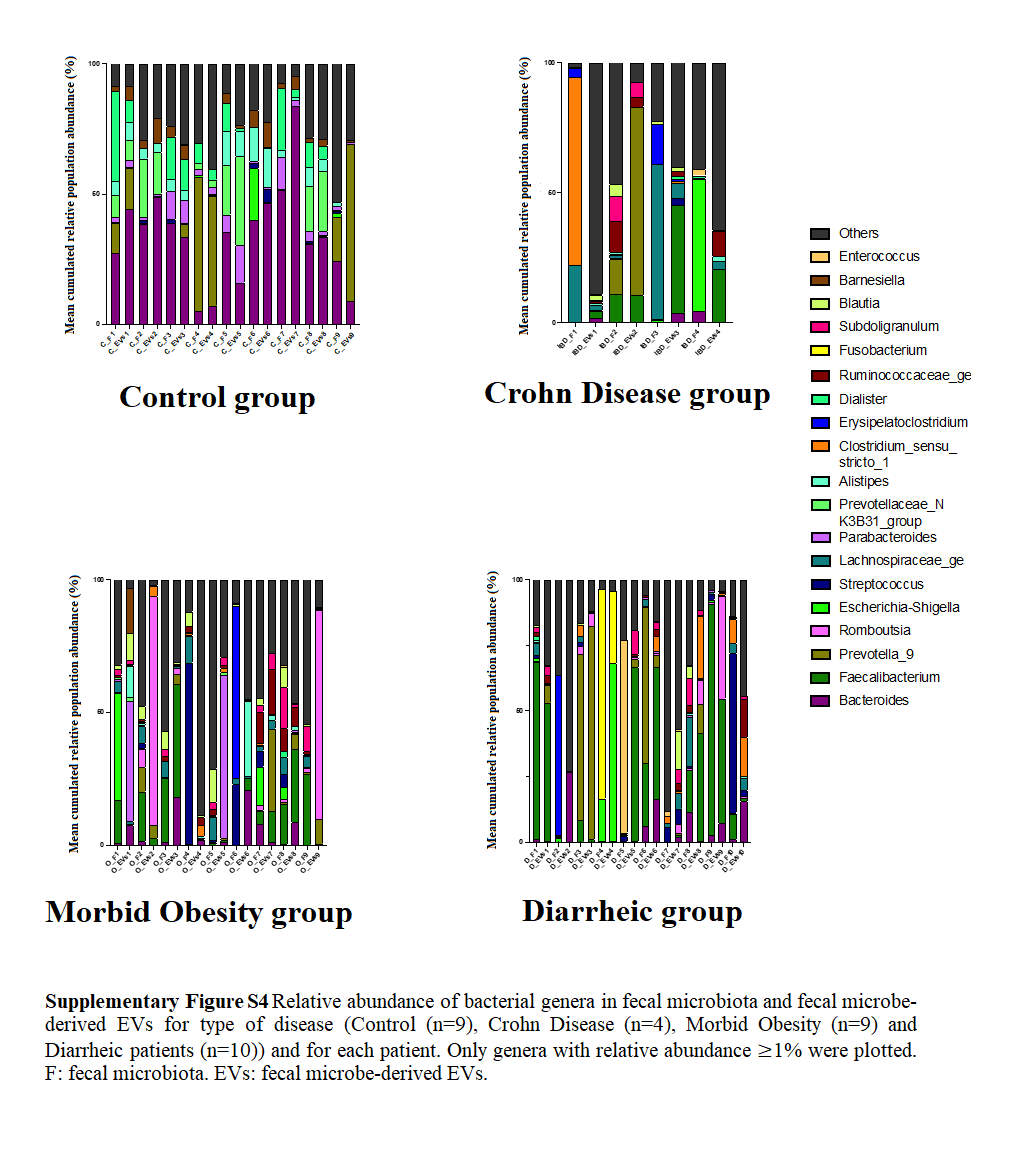

Supplement: Supplementary file 1 [file ijms-24-04971-s001.zip › Supplementary Figure S4.tif]
